# Prediction of Respiratory Irritation and Respiratory Sensitization of Chemicals Using Structural Alerts and Machine Learning Modeling

**DOI:** 10.3390/toxics13040243

**Published:** 2025-03-25

**Authors:** Yaroslav Chushak, Andrew Keebaugh, Rebecca A. Clewell

**Affiliations:** 1 711 Human Performance Wing, Air Force Research Laboratory, Wright-Patterson AFB, OH 45433, USA; 2 Henry M Jackson Foundation for the Advancement of Military Medicine, Inc., Wright-Patterson AFB, OH 45433, USA; 3 BlueHalo, Dayton, OH 45432, USA; 4 EIS, Inc., Wright-Patterson AFB, OH 45433, USA

**Keywords:** respiratory irritation, respiratory sensitization, structural alerts, machine learning modeling, integrative approach

## Abstract

Inhalation of toxic substances and contaminants can have adverse effects on the respiratory tract, leading to a range of health problems, such as irritation and inflammation, allergic reaction and asthma, lung damage, or even death. It is not possible to experimentally evaluate respiratory toxicity for all the thousands of chemicals in use. Here, we generated structural alerts and developed machine learning (ML) classification models to predict respiratory irritation and respiratory sensitization hazards of chemicals using experimental data from publicly available databases and the literature. We identified 13 structural alerts for respiratory irritants and 18 structural alerts for respiratory sensitizers. We also developed a set of models for each hazard using different types of molecular descriptors and ML techniques. Five of the best performing models were combined into a consensus classification model for respiratory irritation, and four individual models were used to develop a consensus classification model for respiratory sensitization. The prediction accuracy of the respiratory irritation consensus model was 84% on the training set and 88% on the test set, and the accuracy of the respiratory sensitization consensus model was 86% on both training and test data sets. A combination of generated structural alerts and ML models was used to screen occupational- and military-relevant chemicals. Out of 687 screened occupational chemicals, 62 compounds were identified as respiratory irritants and 121 chemicals as respiratory sensitizers, while 47 chemicals were predicted as irritants and 36 compounds as sensitizers in the list of 525 military-relevant chemicals.

## 1. Introduction

Inhalation is a common route of exposure to environmental and industrial chemicals. Inhaled substances may cause injury to the respiratory system and induce a wide range of disorders, such as irritation, allergic reaction, asthma, bronchitis, and pulmonary diseases, as well as systemic effects from the absorbed constituents. Recently, we have developed structural alerts and ML models for acute inhalation toxicity (lethality) as a part of a “six-pack” of toxicity endpoints [1]. In the current work, we are focused on site of contact toxicity for inhalation toxicants, specifically respiratory irritation and respiratory sensitization.

Irritant chemicals can cause cell damage and trigger inflammation of the surrounding tissue. One type of respiratory irritation is defined as a localized pathophysiological response to chemicals that involves local tissue redness, swelling, pruritus, and/or pain [2]. Another type of respiratory tract irritation, sensory irritation, is caused by the interaction of the inhaled chemical with the sensory neurons. Respiratory irritant effects in humans include symptoms such as cough, pain, choking, and breathing difficulties. According to the “Globally Harmonized System (GHS) of Classification and Labeling of Chemicals”, which was adopted by the United Nations for chemical hazard classification, chemicals that cause respiratory irritation fall into Category 3 in Specific Target Organ Toxicity (Single Exposure) (STOT-SE), hazard code H335—“May cause respiratory irritation” [3]. Category 3 corresponds to transient target organ effects, i.e., “effects which adversely alter human function for a short duration after exposure and from which humans may recover in a reasonable period without leaving significant alteration of structure or function” [3]. The assessment of chemicals in this category is based on the weight of evidence for respiratory tract irritation, which may include human data and inhalation toxicity studies in animals [3]. Currently, no in vitro models of respiratory irritation have been approved for regulatory use [4].

In contrast to respiratory irritation, respiratory sensitization is an immunological response to a chemical exposure. Development of hypersensitivity requires two phases: induction (or sensitization) and elicitation. In the first phase, the immunological memory is developed in an individual following the initial exposure to a respiratory sensitizer. In the second phase, an antibody-mediated allergic respiratory reaction is elicited following subsequent exposures to a respiratory sensitizer. Hypersensitivity is normally seen as asthma, but it can also be seen as reactions involving the nose (rhinitis), eyes (conjunctivitis), or lungs (alveolitis) [5]. The GHS has a distinct classification for respiratory sensitization, in which chemicals known to induce respiratory sensitization are labeled Category 1. Chemicals are labeled sub-category 1A if there is a high frequency of respiratory sensitization occurrence in humans and sub-category 1B if there is a low to moderate frequency of occurrences. The hazard code for respiratory sensitizers is H334—“May cause allergy or asthma symptoms or breathing difficulties if inhaled” [3].

A significant challenge to assessing respiratory sensitization is the absence of approved animal or in vitro models for the identification and characterization of chemical respiratory allergens. Currently human data are the primary evidence used to identify chemicals as respiratory sensitizers. Because the respiratory sensitization two-phase pattern (induction followed by elicitation) is similar to the development of skin sensitization, test methods used for the evaluation of skin sensitizers may be applied as a surrogate in the weight of evidence during evaluations of potential respiratory sensitizers. In vivo skin sensitization methods include the mouse Local Lymph Node Assay (LLNA) and the Guinea Pig Maximization Tests, both of which have guidelines developed by the Organization for Economic Cooperation and Development (OECD) [6,7]. T cell activation and proliferation in lymph nodes occur with LLNA testing of both dermal and respiratory sensitizers, so the negativity in the LLNA assay could be used to inform which chemicals are unlikely to be respiratory allergens [6]. However, there are some differences in the downstream action of respiratory and dermal sensitizers because of the differing T cell responses, with respiratory sensitizers being associated with T helper 2-type responses and dermal sensitization typically occurring in association with T helper 1 responses [6]. Therefore, some dermal sensitizers are not regarded as respiratory sensitizers, and there are respiratory allergens that do not cause skin sensitization [7]. Thus, positivity in the LLNA assay does not necessarily indicate positivity for respiratory sensitization.

Computational methods, such as structural alerts, read-across, QSAR, and ML modeling, are actively used in risk assessment of chemicals with data gaps [8,9]. Structural alerts are chemical substructures whose presence in compounds is associated with specific types of toxicity. Computational methods for the identification of structural alerts can be divided into two types: expert rule-based approaches and statistical data-driven approaches [10]. Enoch et al. [11,12] used a mechanism-based approach associated with the covalent binding of chemicals to proteins in the lung to identify structural alerts for respiratory sensitization. Using 104 chemicals that caused respiratory sensitization in humans, they identified a set of 52 structural alerts for respiratory sensitization. These structural alerts are used in the OECD QSAR Toolbox (http://qsartoolbox.org, accessed on 20 June 2024) to flag potential respiratory sensitizers [13]. Covalent binding of proteins is a molecular initiating event that is analogous to the molecular initiating event for skin sensitization [6]. To the best of our knowledge, no structural alerts for respiratory irritation have been published.

The existence of databases with large collections of chemical toxicity data allows application of data-driven methods for screening molecular fragments and identifying structural alerts. Several modeling tools have been developed to facilitate this process. One such tool, a Python-based package called SARpy (version 1.3.60), extracts a set of rules directly from data without any ‘a priori’ knowledge [14]. SARpy generates molecular fragments of varying complexity and selects substructures for use as structural alerts based on performance when predicting a training set of chemicals. The resulting structural alerts are a simple and transparent way to flag chemicals as potentially toxic and prioritize them for further evaluation. However, the absence of structural alerts does not imply that a compound is not toxic; it may simply mean that a structural alert has not yet been identified for the compound of interest. Therefore, a strategy that combines structural alerts with QSAR/ML-based predictions of activity or inactivity can significantly expand the applicability domain of computational tools and increase confidence in predictions [9].

The aim of QSAR modeling is to identify patterns in the input data or, in other words, to find correlations between the molecular features of chemicals and their toxicity. ML has the advantage of being able to handle large amounts of data and identify complex relationships. QSAR modeling and ML techniques are widely used today in chemical safety assessment to predict various physico-chemical properties and molecular interactions of chemicals [15,16]. A limited number of QSAR models have been published for prediction of chemical respiratory irritants [17,18,19], while much more attention has been devoted to the development of predictive models for respiratory sensitization. Dik et al. [20] evaluated performance of four commercial and public structure–activity relationship models together with the structural alerts identified by Enoch et al. [12] on a set of newly identified respiratory sensitizers and non-sensitizers. Reported predictivity of the published structural alerts for the new substances was markedly lower than their performance for the chemicals in the original publication, which raises concerns about the utility of the models for novel chemicals [20]. Golden et al. [21] compiled a set of known human respiratory sensitizers to investigate the accuracy of two publicly available computational tools: the ToxTree skin sensitization model [22], and the Centre for Occupational and Environmental Health (COEH)’s occupational asthma model [23]. Both the ToxTree and COEH models achieved respectable accuracy (71% and 72%, respectively), with the COEH model performing better at identifying sensitizers lacking recognized skin sensitization reactivity domains.

In this paper, we describe a combined approach using structural alerts and ML models to predict respiratory irritation and respiratory sensitization of chemicals. The proposed approach was used to predict potential respiratory irritants and respiratory sensitizers in the Occupational Chemical Database compiled by the United States (US) Occupational Safety and Health Administration (OSHA) and in the list of military-relevant chemicals compiled by the US Air Force Research Laboratory. The results obtained indicate the potential of structural alerts in combination with ML models in screening environmental and industrial chemicals for respiratory toxicity, thus creating a valuable tool for accelerating health risk assessment.

## 2. Materials and Methods

**Figure 1:**
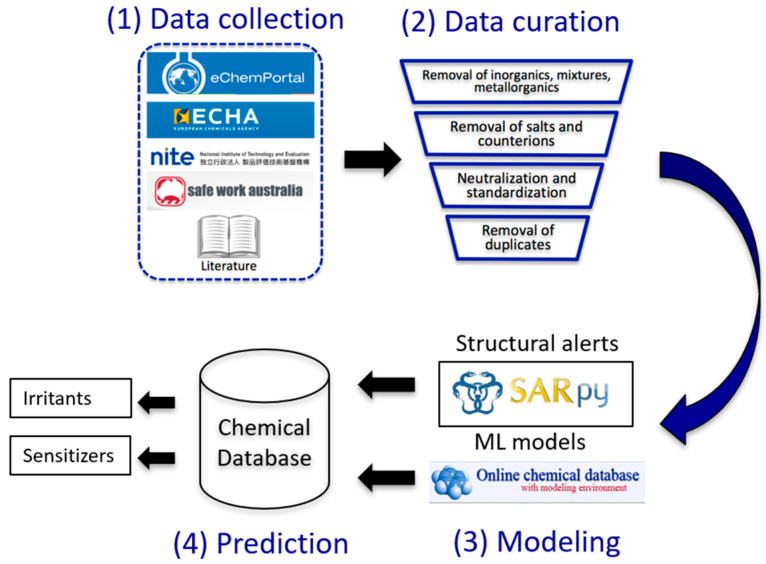
The general workflow of our study includes four steps: (**1**) collection of data from publicly available databases and literature; (**2**) curation and preparation of the compiled data for modeling; (**3**) identification of structural alerts and development of ML models; and (**4**) combined application of structural alerts and ML models to predict potential respiratory irritants and sensitizer.

The current study workflow is presented in Figure 1. Experimental data for respiratory irritation and respiratory sensitization endpoints were compiled from several publicly available databases and published literature sources. After rigorous data curation and cleaning, structural alerts were generated using the SARpy package, and the classification models were developed using the publicly available Online Chemical Modeling Environment (OCHEM, https://ochem.eu). An integrated approach using a combination of predictions from structural alerts and ML models was used to identify potential respiratory irritants and respiratory sensitizers in the lists of occupational- and military-relevant chemicals.

### 2.1. Data Compilation

Data for respiratory irritants were compiled from repositories of three regulatory and authoritative agencies: (1) the Classification and Labeling Inventory (C&L Inventory) of the European Chemicals Agency (ECHA) that provides electronic public access to classification and labeling information on chemical substances placed on the market in the European Union (https://echa.europa.eu/information-on-chemicals/cl-inventory-database, accessed on 6 August 2024); (2) the GHS database of the Japanese National Institute of Technology and Evaluation (NITE) that contains GHS classification results by the Japanese government since 2006 (https://www.chem-info.nite.go.jp/chem/english/ghs/ghs_index.html, accessed on 31 July 2024); and (3) the GHS classification database of the Australian Hazardous Chemicals Information System (HCIS) that contains chemical classification and workplace exposure standards (https://hcis.safeworkaustralia.gov.au/HazardousChemical, accessed on 31 July 2024). The search for respiratory irritants was performed using the STOT-SE class Category 3, which specifically indicates respiratory irritation, or by the hazard code H335. A challenge in this modeling effort was the lack of data for chemicals that have tested negative for respiratory irritation. To collect surrogate non-irritant chemicals, we used data sets that were previously compiled for eye irritation and skin irritation modeling [1]. The data were retrieved from the OECD eChemPortal (https://www.echemportal.org/echemportal/, accessed on 6 August 2024) using the “Property Search” for “Skin Irritation” and “Eye Irritation”, and only included studies that explicitly identified a chemical as “not irritating” in the interpretation of study results. Only data from results categorized as “experimental” and “reliable without restriction” were retrieved.

To collect data for respiratory sensitization modeling, we searched the same three databases by employing the “Respiratory Sensitization” class Category 1, 1A, 1B, as well as hazard code H334. We also gathered data from the OECD eChemPortal using the “Property Search” for respiratory sensitizers and non-sensitizers. For sensitizers we retrieved chemicals with the interpretation of results as “Category 1” or “sensitizing”, whereas for negative compounds the “not sensitizing” labeling was used. If a chemical appeared in both positive and negative categories, such chemicals were labeled as positive because, in the majority of cases, negative results were based on indirect data. Because there were low numbers of chemicals in both categories, “reliable without restriction” and “reliable with restriction” data were used. Additionally, data were compiled from the literature sources [12,20,21] described in the Introduction.

The molecular structures of chemicals were obtained from the Environmental Protection Agency (EPA) CompTox Chemicals Dashboard (https://comptox.epa.gov/dashboard) using Chemical Abstract Service Registry Number (CASRN) identifiers as input (data accessed on 31 July 2024). Obtained Simplified Molecular Input Line Entry System (SMILES) strings were processed through the multistep QSAR-ready workflow in KNIME (version 4.5.2, https://knime.com), which removed mixtures, inorganic compounds, and metallo-organics [24]. The curation procedure included the removal of counterions from salts, the standardization of ring representations, and the neutralization of structures. The structures obtained were converted from SMILES to International Chemical Identifier Key (InChIKey) identifiers using Open Babel software v.3.1.0 (https://sourceforge.net/projects/openbabel/) to remove duplicates.

The list of 687 chemicals in the Occupational Chemical Database, which consists of occupational chemical information compiled by the US OSHA from several government agencies and organizations, was retrieved from the EPA CompTox Chemicals Dashboard. The list of 525 military-relevant chemicals was a subset of a larger military-relevant list composed by the US Air Force Research Laboratory, and the compounds contained were observed at different US Air Force installations [25].

The harmonized functional usage of compounds was obtained by searching the EPA Chemical and Products Database (CPDat) (https://comptox.epa.gov/chemexpo/, accessed on 20 June 2024). CPDat is a database containing reported and predicted information on more than 75,000 chemicals and more than 15,000 consumer products [26].

### 2.2. Generation of Structural Alerts

Structural alerts associated with respiratory irritants and respiratory sensitizers were identified using a Python-based tool called SARpy (https://sourceforge.net/projects/sarpy/). SARpy extracts a set of rules by automatically generating and selecting molecular substructures on the basis of their performance in predicting a training set of active and inactive compounds [14]. The SMILES strings of compounds were fragmented to extract all substructures within a customizable size range. In the present study, a rule was set to identify molecular fragments composed of a minimum of 4 and a maximum of 20 atoms appearing in a minimum of 4 active compounds. SARpy analyzed the correlation between the occurrence of each molecular fragment and the active compounds in the training set. The predictive performance of the resulting molecular fragments as structural alerts was evaluated by calculating the positive predictive value (PPV) as a ratio of true positives to a total number of occurrences. The profiling of structural alerts was performed using the OECD QSAR Toolbox v. 4.5.

### 2.3. Model Development

For ML modeling, the compiled and cleaned data sets for respiratory irritation and respiratory sensitization were uploaded to the OCHEM platform. Data sets were randomly split into training (80%) and test (20%) sets. Initially, ML models were constructed using ten different molecular descriptor sets (“ALogPS, OEstate”, CDK23, Dragon7, Fragmentor, inductive descriptors, JPlogP, Mold2, Mordred, RDKit, ECFP4), which were then processed with the Fast Stagewise Multiple Linear Regression (FSMLR) algorithm [27] for the rapid evaluation of descriptor performance with different data sets. The four best performing descriptors were selected to develop twelve models using Associative Neural Networks (ASNN) [28], Random Forest (RF) [29], and XGBoost ML [30] methods. Additionally, four models were developed using ML algorithms that do not use molecular descriptors, but instead learn molecular features from the representation of molecules as graphs (algorithms implemented within KGGNN [31]) or SMILES (Transformer CNN [32]). All methods were used with default parameters, as specified on the OCHEM website. Finally, the four to five best performing models were used to develop a consensus model for each endpoint using the average of the individual model outputs. Individual models were included into the consensus model if they improved model performance.

### 2.4. Evaluation of Model Quality

The predictive power of the generated models was evaluated using two methods: (1) 5-fold cross-validation of model performance with the training set and (2) accuracy of model predictions with the test set. Three statistical parameters were used to assess the performance of the models based on the confusion matrix consisting of true positive, false positive, true negative, and false negative elements:

$$\mathrm{Accuracy}\;ACC=\frac{TP+TN}{TP+TN+FN+FP}$$

$$\mathrm{Sensitivity}\;SEN=\frac{TP}{TP+FN}$$

$$\mathrm{Specificity}\;SPE=\frac{TN}{TN+FP}$$

Additionally, the area under the receiver operating characteristic curve (AUC) was also used for model evaluation.

### 2.5. Applicability Domain

In OCHEM, the applicability domain of individual classification models was estimated by standard deviation (STD) using the 5-fold cross-validation. For the consensus models, CONS-STD was used to separate reliable and non-reliable predictions. The CONS-STD corresponds to the disagreement (or STD) of the individual predictions of models in the consensus model:

$${CONS-STD}_{j}=\sqrt{\frac{1}{N}\sum_{m=1,\ldots N}{\left(\bar{y_{j}}-y_{j}^{m}\right)}^{2}}$$

where $\bar{y_{j}}$ is the average (consensus) value for molecule *j*, and $y_{j}^{m}$ is a value predicted by *m*-th model [33].

## 3. Results

### 3.1. Data Processing

We compiled respiratory irritants (labeled “active” chemicals) from three different databases: NITE, the ECHA classification and labeling, and HCIS (see Methods and Materials for details). The NITE GHS classification database contained 4090 substances classified as STOT_SE Category 3 class. However, only 642 unique compounds had explicit mentions of “respiratory irritation” in the classification. The molecular structures of these chemicals were obtained from the EPA CompTox Chemicals Dashboard using CASRN identifiers, and only 592 compounds had a valid SMILES string. In a similar way, 389 and 351 substances classified as “active” for H335 hazard code were retrieved from the ECHA classification and labeling database and from the HCIS database, respectively. After cleaning and removing duplicates, the final data set contained 624 chemicals labeled as respiratory irritants. Additionally, a set of 627 chemicals that were classified as non-irritants for both eye and skin irritation were used as “inactive” for respiratory irritation model development.

For the modeling of respiratory sensitization, active chemicals were compiled from the same three databases using the “Respiratory Sensitization” class Category 1, 1A, 1B, or the hazard code H334. The resulting list contained 198 chemicals from the ECHA C&L inventory, 118 chemicals from the NITE GHS classification, and 719 compounds from the HCIS database. However, only 163 compounds in the HCIS database had valid SMILES strings, as the majority of substances were polymers. The OECD eChemPortal “Property Search” for “Respiratory Sensitization” returned 89 sensitizing and 85 non-sensitizing substances. Data were also compiled from the published literature [12,20,21]. Enoch et al. [12] compiled 104 chemicals which have been reported to cause respiratory sensitization, and 82 control chemicals were selected randomly from a list of compounds compiled in the U.K. Health and Safety Executive document EH40, with no indication of cases of occupational asthma reported in the United Kingdom [12]. Dik et al. [20] evaluated the performance of five in silico models for respiratory sensitization on a list of 138 positive compounds and 521 negative chemicals. The majority of the negative chemicals (*n* = 314) were from the EH40 documents, while 167 compounds were derived from LLNA studies with negative results. There is some concern about the application of LLNA results to respiratory sensitization [7,21], so we excluded the chemicals from the LLNA assays as negative compounds for respiratory sensitization. Golden et al. [21] compiled 141 sensitizers and 116 non-sensitizers from various sources. The cleaning and curation of all the compiled data resulted in 208 active chemicals. For non-sensitizers, we additionally performed the profiling of negative chemicals using structural alerts for skin sensitization compiled in the QSAR Toolbox: “Protein binding alerts for skin sensitization according to GHS” and “Protein binding alerts for skin sensitization by OASIS”. Using these structural alerts, we found that 92 chemicals from the initial list of 332 compounds labeled as non-sensitizers had structural alerts for skin sensitization. Additionally, 17 chemicals without the structural alerts were identified as skin sensitizers from in vivo studies. All chemicals with existing structural alerts for skin sensitization or from positive in vivo studies were removed from the list of non-sensitizers. The final list of inactive chemicals for respiratory sensitization modeling contained 211 compounds.

**Figure 2:**
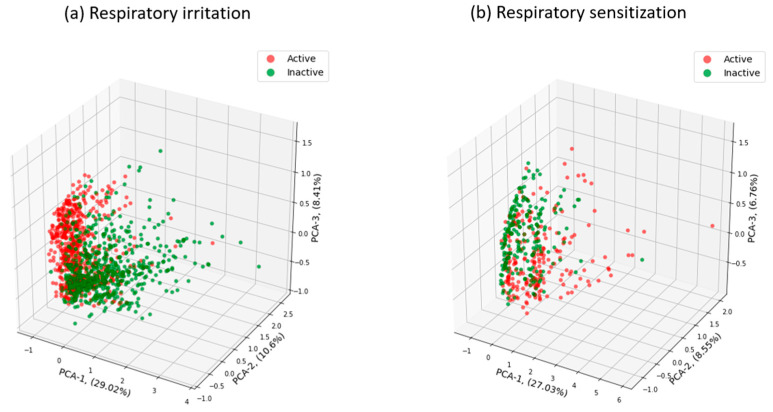
The chemical space distribution of active and inactive chemicals in the respiratory irritation data set (**a**) and respiratory sensitization data set (**b**) calculated by the PCA.

We performed principal component analysis (PCA) of chemical space distribution for active and inactive compounds for irritant and sensitization data sets with chemicals presented using RDKit descriptors. As shown in Figure 2a, active chemicals for respiratory irritation clustered in a relatively localized space while inactive chemicals were widely distributed in the chemical space. There was some overlap between the active and inactive chemicals, and the three major components from the PCA explained ~48% of variables in the respiratory irritation data set. Trends in the PCA for the respiratory sensitization data set were the reverse of the respiratory irritation data set (Figure 2b). For respiratory sensitization, active compounds were widely distributed in the chemical space while inactive chemicals were more localized. Again, there was some overlap between the active and inactive chemicals and the three major PCA components explained ~42% of variables in the respiratory sensitization data set.

### 3.2. Modeling Results

Respiratory irritation. The final data set used for modeling respiratory irritation contained 1241 chemicals, including 624 active chemicals that can cause respiratory irritation and 627 inactive, non-irritant compounds. Using the described workflow, we identified 13 structural alerts for respiratory irritation that were observed in 108 active and 9 inactive chemicals (PPV = 0.92). Obtained molecular fragments were profiled using the OECD QSAR Toolbox to evaluate whether these substructures were already identified as structural alerts for irritation/sensitization (Table 1). Of the structural alerts identified in the current study, nine have been designated as structural alerts for other toxicity endpoints in the OECD QSAR Toolbox. Structural alert #3, which belongs to a class of epoxides, was identified as a structural alert for both respiratory sensitization and skin sensitization. Three molecular fragments (#4—halogenated alkanes, #6—tertiary aliphatic amines, and #8—phenols) were identified as structural alerts for skin irritation in the QSAR Toolbox, and four molecular fragments (#1—anhydrides, #2 and #7—isocyanates, and #10—thiols) were identified as structural alerts for skin sensitization.

**Figure 3:**
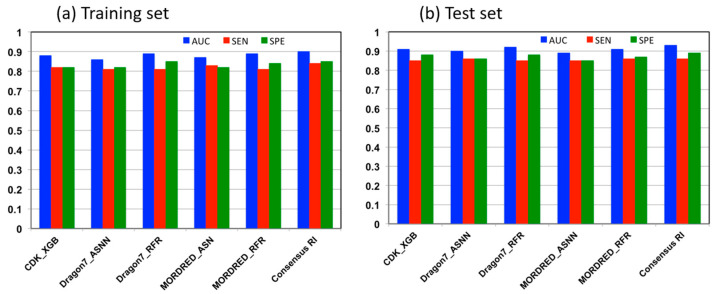
Performance evaluation of five the best individual classification models and consensus model for respiratory irritation on the training set (**a**) and the test set (**b**).

The training set for ML modeling contained 993 molecules, while the test set was composed of 248 compounds. Initially, we evaluated the performance of 10 different molecular descriptors on the training data set using the FSMLR algorithm. Four sets of molecular descriptors (CDK23, Dragon7, MOLD2, and MORDRED) with the best performance on both training and test sets were used to generate models with three ML algorithms: ASNN, RF, and XGBoost. We also developed four models using algorithms that do not require molecular descriptors but learn molecular features employing neural network algorithms. In total, 16 ML classification models were generated. Five models that showed the best performance using all four statistical parameters against the training and test sets were combined to create a consensus model for respiratory irritation. Figure 3 illustrates the performance metrics of the consensus model and the five best individual models that were used for consensus model development on the training and test sets. The complete set of performance metrics for the 10 FSMLR, 16 ML classification, and the consensus respiratory irritation models are presented in the Supplementary Table S1. In general, all models exhibited better performance on the test set compared to the training set, indicating that the model is not overfitted to the training set.

The AUC of the individual models on the training set were in the range of 0.86–0.89, the SEN were in the range of 0.81–0.83, and the SPE in the range of 0.82–0.85. The consensus model that was constructed using these five individual models displayed better performance on the training data than any individual model, with AUC = 0.9, SEN = 0.84, and SPE = 0.85. Evaluation of the individual and consensus models on the test data set showed that the consensus model performed better compared to the best individual models, with AUC = 0.93, SEN = 0.86, and SPE = 0.89 for the consensus model and AUC = 0.91, SEN = 0.86, and SPE = 0.87 for the MORDRED_RFR model. Considering the consensus model’s superior performance on both the training and test data sets, we can conclude that the consensus model offers a more reliable prediction of respiratory irritation.

Respiratory sensitization. The data set for respiratory sensitization modeling was significantly smaller than the data set for respiratory irritation. The modeling set contained 419 chemicals, including 208 sensitizers and 211 non-sensitizers. The application of SARpy to the entire list of chemicals yielded 18 molecular substructures that were observed in 167 sensitizing chemicals and 11 non-sensitizers (PPV = 0.94). The proposed structural alerts for respiratory sensitization and their comparison to structural alerts compiled in the QSAR Toolbox are presented in Table 2. Three molecular fragments (#1—ethanolamines, #2—ethylenediamines, and #6—acrylates) were already identified as structural alerts for respiratory sensitization in the QSAR Toolbox, with the substructure #6 also recognized as a structural alert for skin sensitization. Another three substructures (#4 and #16—aldehydes and #7—isocyanates) were also identified as structural alerts for skin sensitization, with molecular fragments #4 and #16 identified for skin irritation. Five molecular fragments (#3 and #5—aliphatic acids, #8 and #15—phenols, and #10—aromatic amines) were flagged in the QSAR Toolbox as structural alerts for skin irritation. The remaining substructures are potentially novel structural alerts.

**Figure 4:**
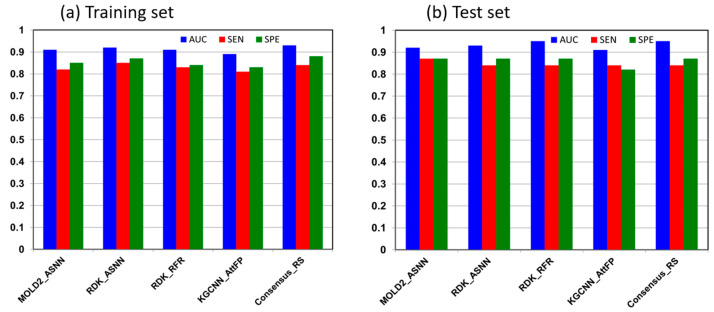
Performance evaluation of the four best individual classification models and the consensus model for respiratory sensitization on the training set (**a**) and the test set (**b**).

The training and test sets for the ML modeling of respiratory sensitization contained 335 and 84 compounds, respectively. Initial screening with the FSMLR method identified CDK23, ISIDA Fragmentor, MOLD2, and RDKIT as the best performing sets of molecular descriptors for the respiratory sensitization data. Of the 16 models developed with these descriptor sets and different ML techniques, four models (MOLD2_ASNN, RDK_ASNN, RDK_RFR, and KGCNN AttFP) showed the best performances on both training and test data sets. These models were selected to generate a consensus model for respiratory sensitization. Figure 4 illustrates the performance evaluation metrics on the training and test sets for the consensus model and the four best individual models that were used for consensus model development. The complete set of performance metrics for the 10 FSMLR, 16 ML classification, and consensus respiratory sensitization models are presented in Supplementary Table S2.

Similar to the respiratory irritation modeling, the performance of ML models for respiratory sensitization was better on the test set compared to the training set of chemicals. The consensus model, which combined predictions of the 4 best individual models, demonstrated similar performance to the RDK_ASNN model (AUC = 0.92, SEN = 0.85, SPE = 0.87) on the training set, with AUC = 0.93, SEN = 0.84, and SPE = 0.88, but better prediction on the test data set, with AUC = 0.93 for the RDK_ASNN model and AUC = 0.95 for the consensus model. On the other hand, the MOLD2_ASNN model showed the best prediction on the test data set, with SEN = 0.87 and SPE = 0.87 but notably lower performance on the training data set, with SEN = 0.82 and SPE = 0.85. This indicates that the consensus model displayed more consistent performance on both training and test sets compared to the individual models, which implies that combining features of different models produces a better overall predictive performance. The consensus approach may be especially important for respiratory sensitization, where the modeling data set is relatively small.

### 3.3. Screening of Chemical Databases for Respiratory Irritants and Sensitizers

**Figure 5:**
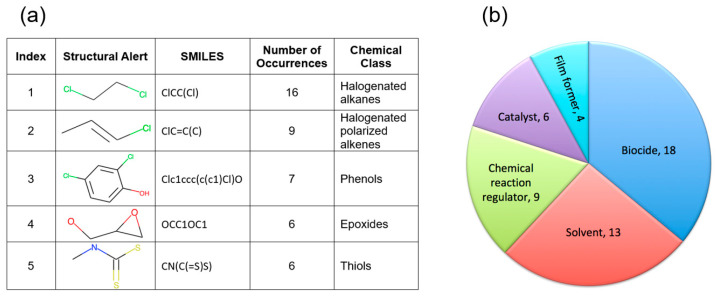
(**a**) Top five structural alerts with the highest number of occurrences observed in 62 irritants identified in the Occupational Chemical Database. (**b**) Top five functional uses of identified irritants.

The combination of generated structural alerts and ML models was used to screen occupational- and military-relevant chemicals. The list of 785 chemicals from the OSHA Occupational Chemical Database was retrieved from the EPA CompTox Chemicals Dashboard (data accessed on 6 August 2024), curated, and cleaned as described in the Methods. The final processed occupational data set contained 687 chemicals. Initially, potential respiratory irritants were identified using the generated structural alerts. A total of 75 compounds contained one or more structural alerts and were labeled as “Active”. The remaining compounds were labeled as “Inconclusive”, as predictions could not be made for chemicals without any structural alerts. Next, the respiratory irritation consensus ML model was employed to identify irritants. The ML model predicted 486 chemicals as “Active”, 161 compounds as “Inactive”, and 40 chemicals were outside the applicability domain and therefore labeled as “Inconclusive”. Next, we calculated a confidence score for each compound using the following scheme: If the compound was predicted as “Active” by the consensus model and contained structural alerts, it was assigned a confidence score of “2”. If one approach predicted compound as “Active”, but another predicted it as “Inconclusive”, a confidence score of “1” was assigned. When both structural alerts and ML model predicted a compound as “Inconclusive”, a confidence score of “0” was allocated. Finally, seven chemicals contained structural alerts but were predicted as “Inactive” by the consensus models. For such chemicals, a consensus score of “−1” was assigned. Combining results from both the structural alerts and the ML consensus model, 62 compounds had a confidence score of “2” and were identified as irritants by the integrated approach, which increased confidence in the classification (see Supplemental Table S3 for detailed results). These 62 chemicals were further analyzed to characterize the processes and substructures contributing to occupational respiratory irritation risks. Out of 13 identified structural alerts for respiratory irritation (Table 1), 12 appeared among the 62 irritants in the Occupational Chemical Database. Only structural alert #13 (chlorosilanes) was absent. Figure 5a lists the five structural alerts with the largest number of occurrences among the identified irritants. It is interesting to note that the top three molecular fragments belong to chlorinated compounds. The functional usage of the 62 identified irritants was obtained from the CPDat database [26]. The most common usage of chemicals predicted to be irritants was in biocides and solvents (Figure 5b). Chlorinated compounds are widely used as antimicrobial agents for disinfection purposes [34] and as solvents in industrial applications [35].

**Figure 6:**
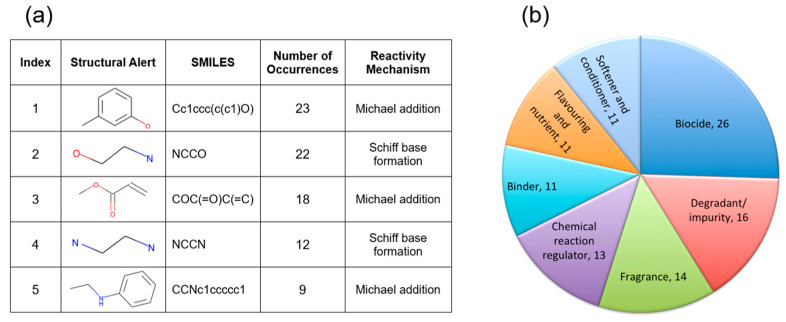
(**a**) Top five structural alerts with the highest number of occurrences observed in 121 respiratory sensitizers identified in the Occupational Chemical Database. (**b**) Top functional uses of identified sensitizers.

Structural alerts and the consensus ML model for respiratory sensitization were also applied to the chemicals compiled in the Occupational Chemical Database. A total of 143 compounds had one or more of the proposed structural alerts for respiratory sensitizers and were identified as “Active”. The consensus ML model predicted 225 chemicals were “Active”, 435 compounds were “Inactive”, and 27 chemicals were outside the applicability domain of the model. Combining structural alerts and the consensus ML predictions resulted in 121 compounds that were likely respiratory sensitizers (see Supplemental Table S4). All molecular fragments identified in this study as structural alerts for respiratory sensitization (Table 2) were observed in the Occupational Chemical Database. The top five structural alerts with the largest number of occurrences among the identified respiratory sensitizers are presented in Figure 6a. Using the QSAR Toolbox, we found that molecular fragments #1, #3, and #5 covalently bind to proteins through Michael addition reactions, and structural alerts #2 and #4 are covalently binding proteins via Schiff base formation. Both mechanisms are common reactions leading to hapten formation, the molecular initiating event in skin and respiratory sensitization [36]. The top functional use of identified sensitizers is presented on Figure 6b. These chemicals are often used in biocides, but they are also observed in fragrances, food products, softeners, and conditioners.

The same combination of structural alerts and ML models was applied to identify respiratory irritants and respiratory sensitizers in a list of military-relevant chemicals compiled by the Air Force Research Laboratory. The integrative approach predicted 47 irritants and 36 sensitizers in a list of 525 chemicals. The results demonstrated that the military-relevant chemicals included about the same number predicted to be respiratory irritants, but significantly fewer chemicals predicted to be respiratory sensitizers compared to the chemicals observed in occupational environment.

## 4. Discussion

Exposure of humans to environmental and occupational chemicals commonly occurs through inhalation and can pose a significant health risk through lung injury or the development of respiratory allergies, particularly when combined with other health risk factors such as respiratory infections and biological susceptibility. While it is never feasible to test all possible toxicants experimentally, respiratory irritants and sensitizers are especially challenging to identify due to the limited availability of test methods accepted by regulatory agencies. Therefore, computational approaches are needed to screen and prioritize chemicals for further evaluation. In this study, we generated structural alerts and applied ML modeling to predict the respiratory irritation and respiratory sensitization of chemicals. The consensus model for respiratory irritation is publicly available on the OCHEM website at http://ochem.eu/model/1176, and the consensus model for respiratory sensitization can be accessed at http://ochem.eu/model/1181. There is a previously published respiratory sensitization consensus model on the OCHEM website https://ochem.eu/article/114857 [37]. However, that model was built using only 136 respiratory sensitizers versus the 208 active chemicals used in our study. Furthermore, the previous model is highly biased toward the inactive compounds, as it predicted active compounds in the training data set with SEN = 0.62 and inactive with SPE = 0.92. It showed better performance with the test data set (SEN = 0.72 and SPE = 0.92). However, the test data set contained only 28 sensitizers. Our consensus model for respiratory sensitization displayed more balanced performance on both active and inactive compounds, with SEN = 0.84 and SPE = 0.88 for the training data set and SEN = 0.84 and SPE = 0.87 for the test data set.

The biggest challenge in the development of computational models for respiratory irritation and respiratory sensitization was data compilation and data cleaning. Because data were retrieved from a variety of databases and published articles, there were many duplicates between sources. The simplest way to identify duplicates was by using CASRN identifiers. However, Enoch et al. [12] provided a list of compounds employed in their study using chemical names and SMILES strings, which are not unique. Therefore, SMILES strings for all compiled chemicals were converted to InChIKey strings to identify and remove duplicates.

Another limitation was the lack of negative data, as the GHS classification that was used to source the positive irritant and sensitization results focused on hazardous chemicals. Negative results are not always published in the literature. Surrogate negative data derived from skin and eye irritation results were used for defining negative respiratory irritants. This route-to-route extrapolation is supported by correlations that have been established between skin/eye irritancy and respiratory irritancy [38,39], as well as shared mechanisms of irritancy between exposure routes, such as transient receptor potential ion channels and direct tissue reactivity [40]. However, the PPV for local irritant respiratory effects by eye and skin irritants (~70–80%) was greater than the negative predictive value (~50–60%) [39]. Similar limitations were encountered in the use of LLNA dermal sensitization data as surrogate negative values for respiratory sensitization. LLNA assays have only been approved for dermal route of exposure, and while there is support for using the assay to identify respiratory sensitizers because of shared early mechanisms of respiratory and dermal sensitization, the divergence in their characteristic downstream immune responses suggest that one is not predictive of the other [6,7]. Furthermore, out of 167 chemicals from the LLNA list evaluated in [20], 138 compounds were predicted as sensitizers by at least one model, with one chemical predicted as sensitizers by four models, 14 chemicals predicted as sensitizers by three models, and 51 chemicals by two models. None of these compounds were used in the development of five models evaluated in [20]. Because we are interested in predicting active chemicals, the lack of the negative data does not preclude the use of the proposed approach, which combines structural alerts and ML modes for screening respiratory irritants and respiratory sensitizers.

Combining structural alerts and ML modeling increases the reliability and confidence in model predictions [9,41]. Out of 62 compounds that were identified as irritants in the Occupational Chemicals Database by integrated approach, 36 of these chemicals were present in the modeling data set, and all were correctly predicted to be irritants by the integrative approach. Similarly, of 121 identified sensitizers in the same database, 35 compounds were present in the modeling set, and all were correctly predicted to be sensitizers. These results indicate that a combination of structural alerts and ML consensus models represents a significant advance toward the goal of NAM-based rapid risk assessment by providing a means to quickly screen and identify potential respiratory toxicants for experimental validation. In vitro models are rapidly being developed, which can provide mechanistic support for computational predictions of lung toxicity. In vitro assays for respiratory sensitization are in developments that use human cells and human reconstructed lung tissue to address key events identified in the adverse outcome pathway [42]. In vitro testing strategies to assess irritant chemicals have also been proposed [43,44].

## 5. Conclusions

We generated structural alerts and developed machine learning models to identify respiratory irritants and respiratory sensitizers. A combination of generated structural alerts and ML models was used to screen occupational- and military-relevant chemicals and to identify potential respiratory toxicants for further evaluation. A testing paradigm that combines in vitro approaches with computational models such as the ones described in this work is likely the best path forward for rapid, non-animal risk assessment of inhaled substances.

## Figures and Tables

**Table 1:** Structural alerts for the respiratory irritants identified in this study and their designation as structural alerts for other toxicity endpoints in the OECD QSAR Toolbox.

Index	Proposed Structural Alert	SMILES	N_Tot_	N_True_	Chemical Class	QSAR Toolbox Alerts
1	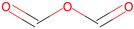	C(=O)OC(=O)	13	12	Anhydrides	Skin sensitization
2	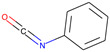	O = C = Nc1ccc(cc1)	13	12	Isocyanates	Skin sensitization
3	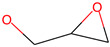	OCC1OC1	13	11	Epoxides	Respiratory/Skin sensitization
4	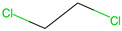	ClCC(Cl)	12	12	Halogenated alkanes	Skin irritation
5	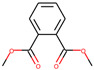	COC(=O)c1ccccc1C(=O)OC	12	8	Phthalate diesters	N/A
6	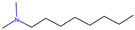	CCCCCCCCN(C)C	11	10	Tertiary aliphatic amines	Skin irritation
7	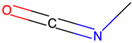	CN = C = O	9	9	Isocyanates	Skin sensitization
8	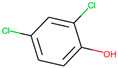	Clc1ccc(c(c1)Cl)O	9	9	Phenols	Skin irritation
9	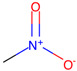	[O-][N+](=O)C	7	7	Nitroparrafins	N/A
10	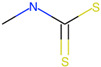	CN(C(=S)S)	6	6	Thiols	Skin sensitization
11	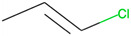	ClC = C(C)	6	6	Halogenated polarized alkenes	N/A
12	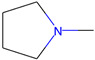	CN1CCCC1	5	5	Aliphatic amines	N/A
13	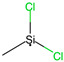	C[Si](Cl)(Cl)	4	4	Chlorosilanes	N/A

The N_Tot_ represents the number of structural alert occurrences in the whole data set, and N_True_ is the number of occurrences in active molecules. Chemical classes are defined by profiling in the QSAR Toolbox.

**Table 2:** Structural alerts for respiratory sensitizers and their profiling with the QSAR Toolbox.

Index	Proposed Structural Alert	SMILES	N_Tot_	N_True_	Chemical Class	QSAR Toolbox Alerts
1	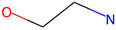	NCCO	36	33	Ethanolamines	Respiratory sensitization
2	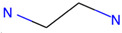	NCCN	28	27	Ethylenediamines	Respiratory sensitization
3	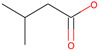	C(C(C)C)C(=O)O	24	24	Aliphatic acids	Skin irritation
4	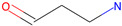	C(=O)C(CN)	23	23	Aldehydes	Skin sensitization, skin irritation
5	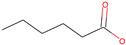	CCCCC(C(=O)O)	18	18	Aliphatic acids	Skin irritation
6	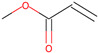	COC(=O)C(=C)	17	17	Acrylates	Respiratory sensitization, skin sensitization
7	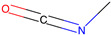	CN = C = O	16	16	Isocyanates	Skin sensitization
8	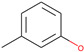	Cc1ccc(c(c1)O)	15	13	Phenols	Skin irritation
9	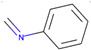	C = Nc1ccccc1	14	14	Primary aromatic amines	N/A
10	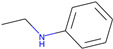	CCNc1ccccc1	13	12	Aromatic amines	Skin irritation
11	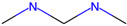	CNCNC	10	8	Aminals	N/A
12	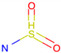	S(=O)(=O)N	9	9	Sulfonamides	N/A
13	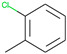	c1cc(C)c(c(c1))Cl	9	8	Halogenated benzenes	N/A
14	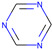	c1ncnc(n1)	8	8	Triazines	N/A
15	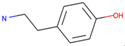	NCCc1ccc(c(c1))O	8	8	Phenols	Skin irritation
16	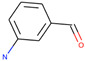	Nc1ccc(c(c1)C(=O))	7	7	Aldehydes	Skin sensitization, skin irritation
17	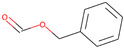	C(=O)OCc1ccccc1	7	6	Esters	N/A
18	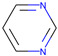	c1ncc(c(n1))	6	6	Pyrimidines	N/A

Notations are the same as in Table 1.

## Data Availability

Data are available in the Supplemental Materials and on the OCHEM web server.

## Supplementary Materials

The following supporting information can be downloaded at: https://www.mdpi.com/article/10.3390/toxics13040243/s1: Table S1 contains performance metrics for 27 respiratory irritation models; Table S2 contains performance metrics for 27 respiratory sensitization models; Table S3 contains results of structural alerts and ML predictions of respiratory irritants in the Occupational Chemicals Database; Table S4 contains results of structural alerts and ML predictions of respiratory sensitizers in the Occupational Chemicals Database.

## Disclaimer

The views expressed are those of the authors and do not reflect the official guidance or position of the United States Government, the Department of Defense, the United States Air Force, or the United States Space Force. The authors are contractors working with Air Force Research Laboratory/711th Human Performance Wing, and the views expressed do not reflect the official guidance or position of Henry M. Jackson Foundation for the Advancement of Military Medicine, Inc.; BlueHalo; or Eagle Integrated Services. The imagery in this document is the property of the United States Air Force. The appearance of, or reference to, any commercial products or services does not constitute DoD endorsement of those products or services.
